# Evaluation of Serum Secretoneurin Levels in Patients With Ischemic Stroke Who Underwent Mechanical Thrombectomy

**DOI:** 10.7759/cureus.36705

**Published:** 2023-03-26

**Authors:** İremgül Güngör, Metin Yadigaroğlu, Çetin K Akpınar, Murat Güzel, Muhammet F Akyüz, Hüseyin T Yanık, Selim Görgün, Murat Yücel

**Affiliations:** 1 Emergency Medicine, Samsun Training and Research Hospital, Samsun, TUR; 2 Emergency Medicine, Samsun University Faculty of Medicine, Samsun, TUR; 3 Neurology, Samsun University Faculty of Medicine, Samsun, TUR; 4 Emergency Medicine, Çarşamba State Hospital, Samsun, TUR; 5 Microbiology, Samsun Training and Research Hospital, Samsun, TUR

**Keywords:** secretoneurin, neurological emergency, mechanical thrombectomy, ischemic stroke, emergency medicine

## Abstract

Background

Ischemic stroke is a focal or global cerebral dysfunction of vascular origin; its treatment aims to provide reperfusion. Secretoneurin is a hypoxia-sensitive biomarker found in high concentrations in brain tissue. We aim to determine secretoneurin levels in patients with ischemic stroke, examine how secretoneurin levels change in the mechanical thrombectomy group, and evaluate the correlation with disease severity and prognosis.

Methods

Twenty-two patients diagnosed with ischemic stroke in the emergency department underwent mechanical thrombectomy, and twenty healthy volunteers were included in the study. Serum secretoneurin levels were measured by the enzyme-linked immunosorbent assay (ELISA) method. Secretoneurin levels were measured at the 0^th^ hour, 12^th^ hour, and 5^th^ day in patients who underwent mechanical thrombectomy.

Results

Serum secretoneurin levels were found to be statistically significantly higher in the patient group (7.43 ng/mL) compared to the control group (5.90 ng/mL) (p=0.023). The secretoneurin levels of the patients who underwent mechanical thrombectomy were 7.43 ng/mL, 7.04 ng/mL, and 8.65 ng/mL, measured at the 0^th^ hour, 12^th^ hour, and 5^th^ day, respectively, and no significant difference was detected in all three time periods (p=0.142).

Conclusion

Secretoneurin appears to be a useful biomarker in the diagnosis of stroke. However, it was found that there was no prognostic value in the mechanical thrombectomy group, and it was not correlated with the severity of the disease.

## Introduction

According to the World Health Organization (WHO), stroke is a rapidly developing, focal, or global cerebral dysfunction of vascular origin that lasts longer than 24 hours or results in death [1]. It can be caused by ischemia or hemorrhage and is one of the common neurological emergencies. Stroke is a common neurological emergency in our country and all around the world [1,2]. Almost all acute stroke patients are first evaluated in the emergency department [3,4]. The main goal of treatment in stroke patients is to provide reperfusion. To this end, intravenous recombinant tissue plasminogen activator (IV rtPA) or mechanical thrombectomy can be applied in suitable patients. Endovascular treatment can be performed with IV rtPA within 4.5 hours from the onset of stroke symptoms or mechanical thrombectomy within six hours for selected patients [5]. Mechanical thrombectomy is superior to medical therapy in acute stroke patients with large vessel occlusion [6].

Secretoneurin is a polypeptide derived from secretogranin-II which is a chromogranin. It includes 33 amino acids [7]. It is secreted from endocrine, neuroendocrine, and neuronal tissues, especially brain tissue. It has been found to have functions such as stimulating striatal neuronal dopamine discharge and activating monocyte migration [8,9]. It is thought to be involved in both neurotransmission and inflammatory processes. Various functions of secretoneurin have also been described, such as angiogenesis, formation of new vessels, growth of neurons, and suppression of melatonin release [10-13]. It has also been detected in much higher concentrations in the cerebrospinal fluid, although it is found in low concentrations in the blood of healthy individuals [12].

Being sensitive to hypoxia and having high concentrations in brain tissue makes secretoneurin a biomarker that can be used in stroke patients. When we examine the current literature; secretoneurin levels have not been previously evaluated in stroke patients undergoing mechanical thrombectomy. Our aim in this study was to evaluate how secretoneurin levels changed in the group with ischemic stroke, assess the diagnostic performance of secretoneurin in the group that underwent mechanical thrombectomy, and investigate its correlation with disease severity and its prognostic value.

## Materials and methods

Our study was approved by the Clinical Research Ethics Committee of Ondokuz Mayıs University on 02.03.2021 with the decision numbered OMUKAEK-2021/27. This study was carried out under the good clinical practices of the Declaration of Helsinki. A detailed informed consent form was filled out for each participant, declaring that they participated in the study.

We have planned this study as a prospective, descriptive study. G*Power 3.1.9.7 was used for the statistical analysis (Heinrich-Heine-Universität Düsseldorf, Düsseldorf, Germany). When the effective width was calculated as d=0.8, alpha error=0.05, and statistical power=0.95 for the “t-test in independent groups,” it was estimated that the study’s sample size should be at least 20 patients. The study was carried out between 01.04.2021 and 30.07.2021 in Samsun Training and Research Hospital Emergency and Neurology Department. During this period, 29 stroke patients over 18 who applied to the emergency department, were diagnosed with ischemic stroke clinically and radiologically, and underwent mechanical thrombectomy due to large vessel occlusion were included in the study. Those under 18 years of age (whose secretoneurin levels might be affected); patients with a history of cerebrovascular disease, cardiopulmonary arrest before or after admission to the emergency department, a history of previous intracranial operation, and a history of epilepsy were not included in the study. Seven patients (two patients with a history of cardiopulmonary arrest, two patients with a history of epilepsy, and three patients with a history of previous cerebrovascular disease) who underwent mechanical thrombectomy within the specified period were not included in the study. Overall, 22 patients were included in the study. In addition, a control group of 20 healthy volunteers was included. Serum secretoneurin levels were measured from the peripheral blood of the patient and control group participants. Serum secretoneurin levels were evaluated in the patient group at the 0th hour before the procedure, the 12th hour, and the 5th day after the procedure.

Peripheral venous blood samples were stored at -80 °C (centigrade degree) until analysis. Samples from all participants were centrifuged at 1500 rpm (rate per minute) for 10 minutes. The commercially available enzyme-linked immunosorbent assay (ELISA) method was used for serum secretoneurin level determination. Secretoneurin determination in serum was performed with the Secretoneurin-Human-ELISA/96 kit (Bioassay Technology Laboratory®, Shanghai, China). Analysis was performed following the instructions given by the manufacturer in the kit package insert. Results were recorded at 450 nm (nanometers) using an ELISA reader (Tecan®, Infinite M200 pro, Austria).

At the same time, in addition to demographic data such as age and gender, occlusion sites, time from symptom onset to recanalization, presence, and type of bleeding, stroke subtype, admission, and 24-hour National Institutes of Health Stroke Scale (NIHSS) scores, Alberta Stroke Program Early Computerized Tomography (ASPECT) scores, Modified Treatment in Cerebral Ischemia (mTICI) score, collateral score and third month modified Rankin Scale (mRS) scores were recorded. Initially, the NIHSS score was used for the clinical evaluation of patients on neurological examination. On day 90, mRS was used to determine the severity of the stroke, detect dependence, and evaluate functional recovery. Good outcomes were defined as mRS 0-2 and bad outcomes as 3-6. Death of the patient within 90 days was defined as mRS [14]. Successful recanalization after treatment was defined as mTICI ≥2b [15].

Statistical analysis

The collected data were recorded by giving numbers to the participants and using the SPSS 24 package program (IBM Corp., Armonk, NY). Analyses were analyzed at a 95% confidence level. To determine the analytic method to be used, since our study group was less than 50 people, the results of the Shapiro-Wilks test were examined, and it was determined whether the data showed a normal distribution. Normally distributed numerical data are expressed as mean ± standard deviation, and non-normally distributed numerical data are expressed as median (minimum-maximum). Mann Whitney U test and Kruskal Wallis H test were used for analyses of numerical data. The chi-square test was used to compare categorical variables. The statistical significance level for all analyzes was accepted as p <0.05.

## Results

A total of 42 cases were included in our study, which included 22 patients who met the criteria versus 20 healthy volunteers. The mean age of the patient group was 70.45 ± 10.33, while the mean age of the control group was 39.40 ± 14.6 (p<0.001). While 59.1% of the patient group (13/22) were female, 30% (6/20) of the control group were female (p=0.114). The clinical features of the patient group are presented in Table 1.

**Table 1:** Clinical Features of Patient Group MCA: Main Cerebral Artery, ICA: Internal Carotid Artery, MCA M1; Main Cerebral Artery Sphenoidal or Horizontal Segment, TANDEM: Internal Carotid Artery and Main Cerebral Artery Occlusion, mTICI Score: Modified Treatment in Cerebral Ischemia Score, mRS: Modified Rankin Score, SAH: Subarachoidal Hemoragia

	Clinical Features of Patient Group	
Clinical Outcome	Alive	17
	Exitus	5
Comorbidities	Hypertension	16
	Coronary Artery Disease	11
	Congestive Heart Failure	6
	Chronic Obstructive Respiratory Disease	3
	Asthma	3
	Chronic Renal Failure	2
	Rheumatoid Arthritis	2
	Atrial Fibrillation	1
Occlusion Site	MCA M1	14
	MCA M1 + ICA	1
	ICA T/L	6
	TANDEM	1
Collateral Score	%100 Good	13
	%50-100 Middle	5
	< %50 Poor	4
mTICI Score	≤ 2A Poor	3
	≥ 2B Good	19
Type of Hemorrage	No Hemoragie	13
	Petechia + Tip 1 hematoma	5
	SAH + Tip 2 hematoma	4
Third-month mRS Score	mRS 0-2	7
	mRS 3-6	15
Subtype of Stroke	Cardioembolic	10
	Cryptogenic	5
	Atherosclerotic disease of large vessels	7

**Table 2:** 0th-hour Secretonorin Levels in the Patient and Control Groups a: Mann Whitney U Test

		n	Median	Minimum	Maximum	U^a^	p
Secretoneurin 0^th ^-hour	Patient	22	7.439	5.080	21.168	129.5	0.023
	Control	20	5.905	5.170	10.40		

The comparison of secretoneurin levels in the patient and control groups is presented in Table 2. Accordingly, serum secretoneurin levels were statistically significantly higher in the patient group than in the control group (p=0.023).

The temporal variation of serum secretoneurin levels (0th hour, 12th hour, 5th day / 7.43, 7.04, 8.65, respectively) was examined. Accordingly, no significant difference was found between the levels of secretoneurin measured at the 0th hour, 12th hour, and 5th day of ischemic stroke patients who underwent mechanical thrombectomy (p=0.142).

The relationship between serum secretoneurin levels and third-month mRS scores of stroke patients who underwent mechanical thrombectomy is presented in Table 3. Accordingly, it was determined that the secretoneurin levels measured at the time of admission and in the following period were not related to the mRS score calculated at the end of the third month (p>0.05).

**Table 3:** Evaluation of Serum Secretoneurin Levels in Stroke Patients According to Third-Month mRS Scores mRS: Modified Rankin Score, ^a^ Mann-Whitney U Test

	3^rd^month mRS	n	Median	Minimum	Maximum	U^a^	p
Secretoneurin 0^th^-hour	Good	7	7.98	6.281	21.168	35.0	0.217
	Poor	15	6.831	5.08	19.986		
	Total	22					
Secretoneurin 12^th^-hour	Good	7	7.689	6.013	18.881	45.0	0.597
	Poor	15	7.027	5.122	14.360		
	Total	22					
Secretoneurin 5^th^-day	Good	7	7.847	5.752	19.446	37.0	0.275
	Poor	15	9.101	5.432	25.915		
	Total	22					

NIHSS scores were recorded in the patient group at admission and the 24th hour and are presented in Table 4. Accordingly, while there was no patient with an NIHSS score of <5 at the time of admission, the number of patients with a 24th-hour NIHSS score of <5 after mechanical thrombectomy was found to constitute 22.7% of all patients, and this difference was found to be statistically significant (p=0.011).

**Table 4:** Admission and 24th-Hour NIHSS in the Patient Group NIHSS: National Institutes of Health Stroke Scale

	Admission NIHSS	24^th^-hour NIHSS	p
NIHSS	Frequency (n,%)	Frequency (n,%)	
<5	0 (0)	5 (22.7)	0.011
5-15	13 (59.1)	12 (54.5)	
16-20	9 (40.9)	5 (22.7)	
Total	22 (100)	22 (100)	

There was no statistically significant difference between NIHSS scores and temporal serum secretoneurin levels in the patient group. There was no statistically significant correlation between the admission NIHSS score and the secretoneurin levels measured at the 0th hour, 12th hour, and 5th day (p=0.077, p=0.0764, p=0.333, respectively). Similarly, no statistically significant correlation was found between the NIHSS score calculated at the 24th hour after mechanical thrombectomy and temporal secretoneurin levels (p=0.956, p=0.394, p=0.326, respectively). There was no significant difference in temporal serum secretoneurin levels between groups with and without bleeding after mechanical thrombectomy (p=0.092, p=0.327, p=0.557, respectively).

## Discussion

In our study, although serum secretoneurin levels were higher in the patient group, no significant difference was found between the serum secretoneurin levels measured at the 0th hour, 12th hour, and 5th day between the groups (p=0.142). It was determined that secretoneurin levels measured at different times were not associated with the mRS score calculated at the end of the third month (p>0.05). There was no significant difference in serum secretoneurin levels between the groups with and without bleeding after mechanical thrombectomy.

Due to the oxygen deficit in hypoxia, secretoneurin levels increase to induce new vessel formation [16-22]. In an animal experiment study; in the hypoxia model created by the obstruction of the carotid arteries, it was observed that the level of secretoneurin increased in neurons in the hippocampus and cerebral cortex [11]. Similarly, increased levels of secretoneurin in muscle cells were observed in the extremity hypoxia model created by femoral artery ligation [16]. In another study; mice with cerebral ischemia were examined. It has been observed that secretoneurin administered intravenously decreases the infarct area and increases the brain’s motor performance and metabolic activity. In this animal model, it was observed that the level of secretoneurin increased in neuronal cells in ischemic areas of the brain. This is similar to post-stroke ischemia in humans. In vitro, secretoneurin reduces apoptosis of neurons in oxygen/glucose deficient cell cultures by stimulating the JAK/STAT pathway [23]. This pathway functions for cytokine receptor signaling, but how the pathway is activated and regulated is not fully understood [24]. In the same study; expression of phosphorylated STAT was found to be significantly increased in ischemic mice treated with secretoneurin, and secretoneurin has been shown to increase vasculature in the ischemic brain region [23]. In another animal study, brain ischemia was induced, and secretoneurin levels were evaluated at the 12th, 24th, 48th hours, and 4th and 7th days. The study observed that the level of secretoneurin increased at the 12th hour, peaked at the 24th hour, and decreased at the 48th hour. On the 4th day, the level of secretoneurin was observed at the same level as the control group and it was observed that it did not change in the following days [25]. In other animal studies, it has been reported that secretoneurin levels increase in the case of cerebral hypoxia, similar to our study [26,27]. Accordingly, secretoneurin may be involved in the pathophysiology of brain ischemia.

In limited clinical studies on humans, it has been shown that secretoneurin levels increase in ischemia states, similar to our study [13,17,21,22,28]. In a study evaluating patients who developed hypoxic brain injury after cardiac arrest, it was observed that the level of secretoneurin increased and was associated with poor clinical outcomes, similar to our study [13]. Another study evaluated 139 newborns, seven of whom were asphyxic. In the study, the level of secretoneurin was measured from the umbilical cord blood. It was observed that the level of secretoneurin in asphyxia newborns was higher in the blood drawn from healthy newborns. Secretoneurin has been thought to be a potential marker of hypoxic-ischemic brain injury in neonates [17]. In a prospective study in 2014, patients admitted to intensive care after successful cardiopulmonary resuscitation were evaluated. In the study, daily secretoneurin levels were measured up to Day 7. serum secretoneurin levels peaked in the first 24 hours, increasing to six times their normal level. The neurological prognosis of the patients was estimated with the Cerebral Performance Categories Scale (CPC). Patients with high levels of secretoneurin and high CPC scores have poor neurological outcomes. It has been shown that it can be used in the early recognition of hypoxic brain injury due to its predictable prognosis because secretoneurin increases in the first 24 hours and is associated with high CPC scores [13].

Since the ischemic area may develop in a larger area in strokes due to large vessel occlusion, higher serum secretoneurin levels can be expected. The effect of collateral circulation on clinical improvement and its relationship with less infarct volume has been shown [29]. Since the infarct volume is expected to be less in cases whose vessels are opened by mechanical thrombectomy, patient-specific variability in serum secretoneurin levels can be observed. In other words, serum secretoneurin levels can be expected to increase at a lower rate in this group of patients compared to the untreated group.

In our study, secretoneurin levels decreased in the group with an mRS score of 0-2 at the 12th hour, and a small increase was observed again on the fifth day. Secretoneurin levels increased continuously with an mRS score of 3-6. However, there was no statistical significance between the serum secretoneurin levels and the third-month mRS scores groups [4]. According to our results, in patients with a smaller mRS score and less disability, a decrease in secretoneurin levels was observed after mechanical thrombectomy, probably because the area of ischemia was smaller. In patients with a higher mRS score and poor prognosis, secretoneurin levels increased independently of the width of the ischemia area or mechanical thrombectomy.

There are limitations to our study due to a few factors. It was a single-center study with a small number of patients. The patients in our study are a special group and mechanical thrombectomy cannot be applied to many stroke patients, considering the additional disease, admission time, and independence levels, so our sample size is limited. In addition, our patient group who underwent mechanical thrombectomy was very heterogeneous, and many factors affected the clinical outcome. The mean age of the patient and control groups was heterogeneous, which may have affected the results of age-related secretoneurin levels. We found that secretoneurin levels were increased in stroke patients, but we could not show its relationship with mechanical thrombectomy. Different results can be obtained by conducting multicenter studies in which patients with stroke are compared with or without mechanical thrombectomy, or involving a large number of patients.

## Conclusions

We showed that ischemia-induced serum secretoneurin levels increase in stroke patients, but we could not show the relationship of this situation with mechanical thrombectomy. In our study, although secretoneurin levels are a useful diagnostic biomarker in stroke patients, we could not show a relationship between neurological outcomes or prognosis.
